# Overexpression of *SoCYP85A1*, a Spinach Cytochrome p450 Gene in Transgenic Tobacco Enhances Root Development and Drought Stress Tolerance

**DOI:** 10.3389/fpls.2017.01909

**Published:** 2017-11-09

**Authors:** Fangmeng Duan, Jun Ding, Dongsun Lee, Xueli Lu, Yuqi Feng, Wenwen Song

**Affiliations:** ^1^College of Plant Health and Medicine, Qingdao Agricultural University, Qingdao, China; ^2^Key Laboratory of Analytical Chemistry for Biology and Medicine, Ministry of Education, Department of Chemistry, Wuhan University, Wuhan, China; ^3^College of Applied Life Science, Jeju National University, Jeju, South Korea; ^4^Marine Agricultural Research Center, Tobacco Research Institute, Chinese Academy of Agricultural Sciences, Qingdao, China; ^5^Wuhan Institute of Biotechnology, Wuhan, China

**Keywords:** drought stress tolerance, transgenic tobacco, *SoCYP85A1*, brassinosteroids, stress-responsive genes, ROS

## Abstract

Brassinosteroids (BRs) play an essential role in plant growth, development, and responses to diverse abiotic stresses. However, previous studies mainly analyzed how exogenous BRs influenced plant physiological reactions to drought stress, therefore, genetic evidences for the endogenous BRs-mediated regulation of plant responses still remain elusive. In this study, a key BRs biosynthetic gene, *SoCYP85A1* was cloned from *Spinacia oleracea*, which has a complete open reading frame of 1,392 bp encoding a 464 amino acid peptide and shares high sequence similarities with CYP85A1 from other plants. The expression of *SoCYP85A1* which was higher in leaf compared with root and stem, was induced by treatments of PEG6000, abscisic acid (ABA), low temperature and high salt. Increases in both *SoCYP85A1* transcripts and endogenous BRs in transgenic tobacco which resulted in longer primary root and more lateral roots enhanced drought tolerance compared with wild types. The transgenic tobacco accumulated much lower levels of reactive oxygen species and malondialdehyde (MDA) than wild types did, accompanied by significantly higher content of proline and notably enhanced activities of antioxidant enzymes. Besides, transcriptional expressions of six stress-responsive genes were regulated to higher levels in transgenic lines under drought stress. Taken together, our results demonstrated that *SoCYP85A1* involves in response to drought stress by promoting root development, scavenging ROS, and regulating expressions of stress-responsive genes.

## Introduction

Abiotic stress such as drought, has been a primary factor adversely affecting plants quality and production in the natural environment (Farooq et al., 2009; Li et al., 2009). Plants have evolved intricate defense mechanisms to cope with water loss, which might involve physiological or molecular processes by regulating concentrations of phytohormones (Nemhauser et al., 2006; Gruszka, 2013; Xia et al., 2014) and expression of stress-related genes (Kodaira et al., 2011; Wang et al., 2015; Udawat et al., 2016).

Brassinosteroids as one of the most important phytohormones involve in various physiological processes (Choudhary et al., 2012). In addition of their roles in plant growth and development (Hu et al., 2000; Arteca and Arteca, 2001; Yu et al., 2004), BRs have recently been implicated in response to abiotic stresses (Kagale et al., 2007; Xia et al., 2009; Chung et al., 2014; Li et al., 2016a). Most previous studies focused on BRs regulations on plant response to abiotic stresses have been performed using exogenous BRs (Kurepin et al., 2008; Li and Feng, 2011; El-Mashad and Mohamed, 2012), however, the genetic mechanisms for endogenous BRs in response to stresses remain poorly understood (Chung et al., 2014; Zhou et al., 2014; Divi et al., 2016).

Brassinosteroids biosynthetic pathway has been annotated clearly by using GC-MS and genetic modified analysis in the last few years. To date, key enzymes such as DWF4, CPD, DET2, CYP90D1, CYP85A1, and CYP85A2 have been characterized to catalyze the conversion of the intermediates in the BRs biosynthesis pathway (Fujioka et al., 1997; Shimada et al., 2003; Kim et al., 2005; Ohnishi et al., 2012), which were all found to encode cytochrome P450 monooxygenases. Since the first BRs, BL was discovered in 1979 (Grove et al., 1979), over 70 BRs have been isolated and characterized (Tarkowská et al., 2016). Applications of exogenous BRs to plants result in various physiological effects, including promotion of germination and cell elongation, differentiation of tracheary element, enhancement of photomorphogenesis, pollen fertility and stress resistance (Clouse and Sasse, 1998; Steber and McCourt, 2001).

A number of BRs-deficient mutants in absence of BR biosynthesis genes represent typical characteristics with rounded leaves, shortened petioles, reduced fertility and delay in senescence (Vert et al., 2005). Similarly, BRs-deficient mutants in absence of BRs receptors exhibit the same abnormalities in the BRs metabolism, which demonstrated that BRs receptors are involved in BR signal transduction pathways. BRs signals are detected by BRs receptors such as BRASSINOSTEROID INSENSITIVE 1 (BRI1) in the cell membrane (Li and Chory, 1997; Wang et al., 2002). Within the past two decades, more than 20 different types of BRI1 mutants have been identified. Most of these weak mutants served as genetic screening materials, have made important contributions to BL synthesis, metabolism and signal transduction. It was recently reported that overexpression of *GmBRI1b* repressed the relatively high expression of genes involved in BRs biosynthesis, including *DWF4*, *CPD*, *CYP85A1*, and *CYP85A2* in the transgenic *bri1*-5 mutant (Peng et al., 2016). A number of BRs signaling regulators, such as, BAK1 (Li et al., 2002), BRS1 (Li et al., 2001), BEN1 (Yuan et al., 2007), TCP1 (Guo et al., 2010) have been characterized by screening genetic suppressors of *bri1*-5 via the activation-tagging method. In detail, BAK1 and BRS1 have been confirmed to promote BRs signaling by interacting with and phosphorylating BRI1. *BEN1* is responsible for BRs metabolism and overexpression of this gene could enhance the dwarf phenotype of *bri1-5* (Yuan et al., 2007). *TCP1* positively coordinates with the function of DWF4, a key enzyme in BRs biosynthesis, suggesting another level of regulation through which BRs mediate plant growth and development (Guo et al., 2010).

Since BRs biosynthetic pathway was analyzed gradually clearly, researchers have carried out to explore the biological functions of endogenous BRs via regulating expressions of related genes involved in BRs biosynthesis. Overexpression of *CYP85A1* in tomato which increased the contents of 28-norCS and BL promoted seed germination, enhanced carotenoid content (Li et al., 2016a) and improved photosynthetic efficiency (Li et al., 2016b). These above reports mainly focused on plant growth and development influenced by overexpression of *CYP85A1*, however, researches on relationship between abiotic stress and *CYP85A1* remained lack. Although physiological features between wild types of barley and semi-dwarf mutants of *CYP85A1* were compared under drought stress, genetic mechanisms had not yet been revealed (Janeczko et al., 2016). In another recent report, overexpression of the BRs biosynthetic gene *CYP90B1* in Brassica napus increased stress tolerance by regulating expressions of dehydration responsive genes (Sahni et al., 2016). However, genetic mechanisms how the *CYP85A1* gene participates in plant response to drought stress remain still unknown.

There have different biosynthetic pathways of BRs among different plant species. In *Arabidopsis* and tomato, there are two pathways to biosynthesize CS, part of which is catalyzed to BL. While there is only one pathway in tobacco and rice, in which a small amount of CS turn into 3-epiCS (Fujioka et al., 1997; Hong et al., 2005). Besides, CYP85A1 is an enzyme to catalyze intermediate metabolites to produce CS (Shimada et al., 2001). Therefore, in order to explore the function of *CYP85A1* and CS at the genetic level, we cloned this gene from spinach and generated *CYP85A1*-overexpressing tobacco. It was demonstrated that overexpression of *CYP85A1* enhanced drought tolerance and root development of transgenic tobacco compared with wild types. In addition, we identified the molecular mechanism that *CYP85A1* enhanced drought tolerance by eliminate ROS accumulation and modulating expression of stress-responsive genes.

## Materials and Methods

### Plant Materials and Stress Treatments

Spinach (*Spinacia oleracea* L.) seeds were sterilized and cultured in growth chambers under the condition of 16 h light/8 h dark cycle at 20°C with 50% relative humidity for 14 days. The materials were collected from the root, stem, and leaf, respectively, under the normal condition. For drought, salt or ABA treatment, the shoots were transferred to beakers containing fresh distilled water added with 20% PEG6000, 200 mM NaCl or 100 μM ABA, which were kept in the same growth chamber for designated time (0, 1, 3, 6 12, 24, and 48 h for drought and ABA stress; 0, 1, 3, 6 12, and 24 h for salt stress). Low temperature stress was carried out by transferring shoots to 4°C for 0, 1, 3, 6 12, 24, 48, and 72 h. For each treatment, 10 shoots were used, among which, 3 shoots were randomly selected at each time point. The materials from all treatments were frozen immediately in liquid nitrogen and stored at -80°C for RNA extraction.

### Cloning and Bioinformatics Analysis of the *SoCYP85A1* Gene

Total RNA was extracted from plant leaves using the Easy-BLUE kit (Intron Biotech, Korea). Reverse transcription to synthesize the first strand cDNA was performed with Maxime^TM^ RT PreMix Kit (Intron biotech, Korea). Full-length cDNA of *SoCYP85A1* was generated by rapid amplification of cDNA ends (RACE) technique using Gene-RACE Kit (Invitrogen, United States) according to the manufacturer’s instructions. The specific primers and nested primers were designed by the Primer 5.0 program based on the conservative sequences of *CYP85A1* in other plants (**Table 1**). The PCR amplifications were performed using *Ex-Taq* DNA polymerase (Takara, Japan) with the template of the first strand cDNA. The PCR program consisted of a 5-min incubation at 94°C, 30 cycles of 30 s at 94°C, 30 s at 65°C, and 90 s at 72°C, followed by a 10-min extension at 72°C. The PCR product was purified by a Gel Extraction Kit (QIAGEN, Hilden, Germany) to ligate sub-cloned into the pMD18-T vector (TaKaRa, Japan) and then sequenced.

**Table 1:** Sequences of specific primers and nested primers in cloning *SoCYP85A1.*

Description	Sequences (5′–3′)
Gene specific primer for *CYP85A1* 5’-RACE	GGTTAGTACCAGGGTACCCACAGTC
Gene specific nested primer for *CYP85A1* 5’-RACE	GGGGTGTCCAACAATAGTGTC
Gene specific primer for *CYP85A1* 3’-RACE	AGAAGGGCACCCCTCATGTACT
Gene specific nested primer for *CYP85A1* 3’-RACE	GTTGGGCAGTTGAGCTTGATT
Gene RACE 5’ primer	CGACTGGAGCACGAGGACACTGA
Gene RACE 5’ nested primer	GGACACTGACATGGACTGAAGGAGTA
Gene RACE 3’ primer	GCTGTCAACGATACGCTACGTAACG
Gene RACE 3’ nested primer	CGCTACGTAACGGCATGACAGTG


The full-length cDNA of *SoCYP85A1* was analyzed by the BLAST algorithm^1^. Multiple alignments were carried out using the DNAMAN software between the deduced amino acid sequence of *SoCYP85A1* and other *CYP85A1* homologs from different plant species obtained from NCBI. The phylogenetic relationship tree was constructed by the neighbor-joining method using MEGA (version 7.0). Theoretical molecular weight and isoelectric point (pI) and were calculated with ProtParam tool^2^.

### Analysis of the *SoCYP85A1* Gene Expression by Quantitative Real-time PCR (qRT-PCR)

qRT-PCR was performed to evaluate expression levels of *SoCYP85A1* under different treatments. RNA isolation (QIAGEN, Hilden, Germany) and cDNA synthesis (Takara, Japan) were performed based on the manufacturer’s instructions. The cDNA solution was used as templates for PCR amplifications with two pairs of specific primers of the *SoCYP85A1* gene and *Spinacia oleracea* actin gene (GenBank: JN987183.1) as an internal control (**Table 2**).

**Table 2:** Sequences of primers in qRT-PCR.

Genes	Sequences (5′–3′)	
	Forward	Reverse
*SoCYP85A1*	AATCAAGCTCAACTGCCCAAC	CAGGGAGGTCAATAGGGAGAGA
*Actin*	GATTCTGGTGATGGTGTTAGT	CTCCGATTGTGATGACTTGT
*GFP*	AGCTGACCCTGAAGTTCATCTG	ACTGGGTGCTCAGGTAGTGGTT
*Bar*	CTCTAGGGGTCATCAGATTTCG	CAACCACTACATCGAGACAAGC
*NtNCED1*	CTATTTCCACTTCAAAACCAACCAC	GGCACTTTCCACGGCATCT
*NtADC1*	GGGAGGTAATGTTGGGGTTTG	TTTTGAGCAGCCGAGGTGT
*NtSAMDC*	ATTGGTTTTGAAGGTTTTGAGAAG	TCACGTCTTGTACTTTGAGAGACAG
*NtAPX*	CAAATGTAAGAGGAAACTCAGAGGA	AGCAACAACTCCAGCTAATTGATAG
*NtCAT*	AGGTACCGCTCATTCACACC	AAGCAAGCTTTTGACCCAGA
*NtGST*	GGCAACAAAAGGAGAAGAGCA	CCAATCAGAGCAATATCCACAAAC
*NtSOD*	CCGTCGCCAAATTGCATAG	CGATAGCCCAACCAAGAGAAC
*NtERD10C*	ACGTGGAGGCTACAGATCGTGGTTTG	TCTCCACTGGTACAGCCGTGTCCTCAC
*NtLEA5*	GAACCCAACAAGAGCGAGAGA	CGACAGGAAGCATTGACGAG
*NtActin*	CAAGGAAATCACCGCTTTGG	AAGGGATGCGAGGATGGA


qRT-PCR was conducted in a Real-time System (qTOWER Applied Biosystems, Jena, Germany) using SYBR Green PCR Master Mix (Takara, Japan) with following PCR program, 95°C for 5 min, followed by 40 cycles of 95°C for 15 s, and 62 (GSP2)/60 (Actin)°C for 1 min. The relative level of gene expression was calculated through the 2^-ΔΔCt^ formula. Each sample was amplified by three biological replicates.

### Transformation of Tobacco and Regeneration of Transgenic Plants

T_0_ generate transgenic tobacco plants expressing SoCYP85A1-GFP fusion protein, the target gene was inserted in the vector pB7WG2D, 1-GFP to create expression vector under the control of CAMV35S promotor and NOS terminator, which was transferred into *Agrobacterium* strain LBA4404. Transgenic tobacco plants were generated via the leaf disk transformation method (Horsch et al., 1985).

T_0_ transgenic seeds were germinated on MS medium containing glufosinate (5 mg/L). Putative transgenic plants were confirmed by PCR amplifications of the *SoCYP85A1* gene to select T_2_ and T_3_ seeds. Ten lines were scanned by qRT-PCR to detect expressions of the *SoCYP85A1* gene at the transcriptional level.

### Assay for Drought Tolerance

Seeds of the three types as described above were cultured in pots containing the same amount of soil in growth chambers under the condition of 16 h light/8 h dark cycle at 28°C with 60% relative humidity. Six-week-old WT and transgenic lines were withheld water for 10 days to compare the drought tolerance. The leaves of 6-week-old seedlings exposed to water stress for 10 days were sampled to measure endogenous BRs, ABA, physiological indexes, and gene expressions.

### Extraction and Quantification of Endogenous BRs

Quantification of endogenous BRs was performed as described previously (Ding et al., 2013).

### Extraction and Quantification of Endogenous ABA Content

Quantification of endogenous ABA was carried out according to the following steps. In brief, about 0.1 g samples were grinded and then added 1 mL precooling reagent 1 at 4°C overnight. Samples were centrifuged at 8000 ×*g* for 10 min. Afterward, the supernatant was transferred into another tube and dried with nitrogen until there was 0.5 mL solution left, in which 0.5 mL reagent 2 was added to extract and decolored for three times. The upper ether phase was discarded and the lower one continued to be dried, which was dissolved in 0.5 mL methanol, filtered and used for HPLC analysis.

The samples were all measured on the HPLC apparatus (e2695, Waters, United States) coupled with reversed phase column of Kromasil C18. The flowing phase was consisted of methanol and water (2:3 v/v). The injection volume was 10 μL, of which flowing rate was 0.8 mL/min at column temperature of 35°C and wave length of 254 nm for 40 min.

### Measurements of RWC, Water Loss Rate, Contents of H_2_O_2_, Proline, MDA, and Activities of Antioxidant Enzymes

Relative water content and water loss rate were measured as previous reports described (Du et al., 2010; Udawat et al., 2016). Contents of H_2_O_2_, proline, MDA, and activities of antioxidant enzymes (SOD, POD, and CAT) were spectrophotometrically determined with detection kits (H202-1-Y, A107, A003-3, A001-4, A084-3, A007-1, Jiancheng, Nanjing, China) according to the manufactural instructions.

### qRT-PCR Analysis of the Downstream Genes Regulated by *SoCYP85A1*

The samples used for this assay were collected before and after treatments as described for drought/salt stress assay. RNA isolation and cDNA synthesis were performed as mentioned above. qRT-PCR was utilized to examined transcriptional levels of nine genes encoding enzymes involved in ABA biosynthesis (*NtNCED1*), polyamine biosynthesis (*NtADC1* and *NtSAMDC*), ROS detoxification (*NtAPX*, *NtCAT*, *NtGST*, and *NtSOD*), as well as stress-responsive proteins (*NtERD10C* and *NtLEA5*). The *NtActin* gene was used as the internal control. The sequences of primers for qRT-PCR were list in **Table 2**.

### Statistical Analysis

The experiments were conducted for three times, each of which contained three biological replicates. Data presented as the mean ± SD were analyzed by SPSS statistical software (ver.16.0, SPSS Inc., Chicago, IL, United States) via one-way analysis of variance. A Tukey’s test (*P* < 0.05) was applied for the significant difference statistical analysis.

## Results

### Cloning and Bioinformatics Analysis of *SoCYP85A1*

In this work, a new *CYP450* gene was isolated by RACE from spinach and designated as *SoCYP85A1* (GenBank Accession: KT900949). Sequence analysis presented that the *SoCYP85A1* cDNA is 1669 bp with a 1392 ORF encoding a deduced protein of 464 amino acids with molecular weight of 54 kDa and isoelectric point of 9.66. Conserved domain of the SoCYP85A1 protein was analyzed and indicated that it belongs to p450 superfamily (Supplementary Figure 1A). Multiple alignments showed that the SoCYP85A1 protein presented a high sequence identify with other CYP85A1 proteins from *Fagopyrum esculentum* (BAO79854, 81%), *Morus notabilis* (XP_010099314, 80%), *Arabidopsis thaliana* (NP_851105, 80%), *Medicago truncatula* ((XP_013451102, 79%), *Theobroma cacao* (XP_007032850, 79%), *Glycine soja* (KHN08349, 78%), *Nicotiana tabacum* (NP_001312136, 78%) and *Solanum lycopersicum* ((NP_001234263, 76%) (Supplementary Figure 1B). The phylogenetic analysis revealed that SoCYP85A1 had a closer relationship with the CYP85A1 protein from *Fagopyrum esculentum* than those from other plant species (Supplementary Figure 1C).

### Expression Patterns of *SoCYP85A1* under Stress Treatments

**FIGURE 1:**
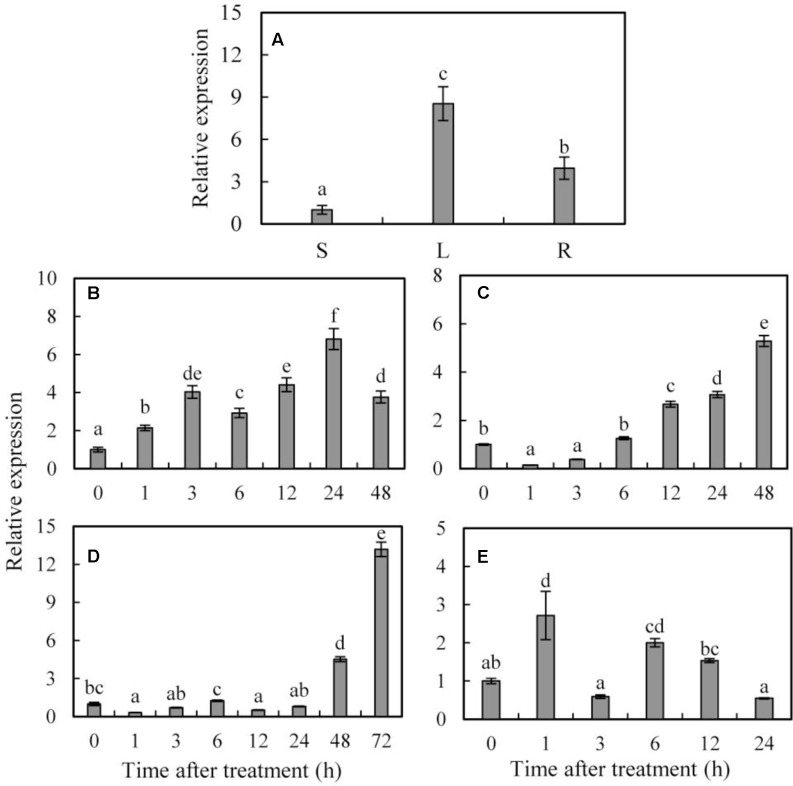
Organ expression assay of *SoCYP85A1* and expression profiles of *SoCYP85A1* under treatments with PEG, ABA, 4°C and NaCl. **(A)** Organ expression assay of *SoCYP85A1* in spinach. The organs (stem, leaf, and root) are represented by S, L, and R, respectively. **(B)** 20% PEG6000; **(C)** 100 μM ABA; **(D)** 4°C; **(E)** 200 mM NaCl. The expression level of stem for organ expression assay and for stress assay at time point 0 were defined as 1. Error bars represent standard deviations (SD) for three independent replicates with different letters at *P* < 0.05 significant level.

qRT-PCR was performed to determine the expression patterns of *SoCYP85A1* in different tissues under normal condition or various abiotic stresses. The data showed that expression of *SoCYP85A1* was detected in all three tissues, which was higher in leaf compared with stem and root (**Figure 1A**).

The transcript level of *SoCYP85A1* began to accumulate 1 h after PEG6000 treatment and reached maximum at 24 h, which was 6.81-fold of the initial level followed by a decrease at the end of the treatment (**Figure 1B**). When subjected to ABA stress, the *SoCYP85A1* abundance was reduced in the first 3 h but gradually increased by 5.28-fold at 24 h (**Figure 1C**). Low temperature treatment led to a slight down-regulation first but a significant up-regulation by 4.52-fold at 24 h and 13.18-fold at 48 h, respectively (**Figure 1D**). During the high salt treatment, the transcription of *SoCYP85A1* performed a slight increase and at the first 1 h, which was only 2.71-fold of that at 0 h (**Figure 1E**).

### Regeneration of Transgenic Tobacco Plants Overexpressing *SoCYP85A1*

**FIGURE 2:**
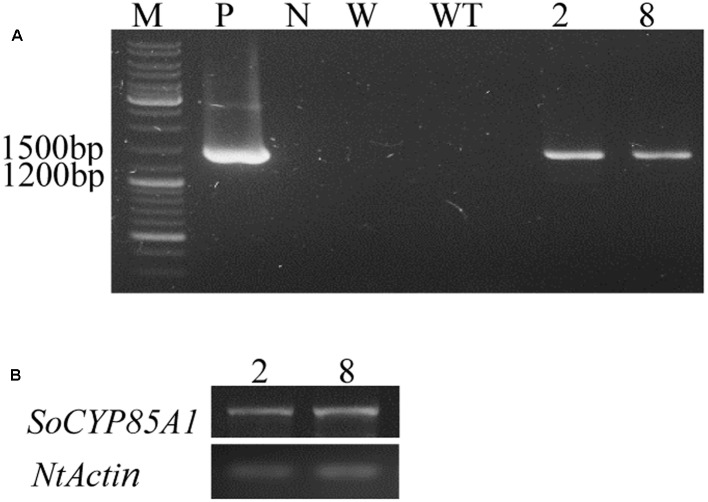
Molecular identifications of transgenic tobacco lines. **(A)** PCR analysis of genomic DNA with the specific primers for the *SoCYP85A1* gene. PCR amplification of a 1,392 bp fragment of the *SoCYP85A1* gene in transgenic lines. **(B)** Expressions of *SoCYP85A1* in transgenic lines. M, molecular marker; P, plasmid; N, negative control; W, water; WT, wild types; 2 and 8, transgenic lines.

The ORF of *SoCYP85A1* was overexpressed in tobacco under the control of 35S promotor of cauliflower mosaic virus (CaMV 35S). Two lines of T_3_ generation were confirmed by PCR analysis of genomic DNA with the specific primers for the *SoCYP85A1* gene (**Figure 2A**). qRT-PCR was carried out to analyze expression levels in the two lines (**Figure 2B**). The WT and two lines (L2 and L8) were exposed to drought treatment to investigate the function of *SoCYP85A1*.

### Overexpression of *SoCYP85A1* Regulates Root Development and Enhances Drought Tolerance

Root development was changed between WT and the two transgenic lines at the stage of 6-week-old under the regular irrigation. Compared with WT, transgenic lines showed longer primary root and increased number of lateral root.

**FIGURE 3:**
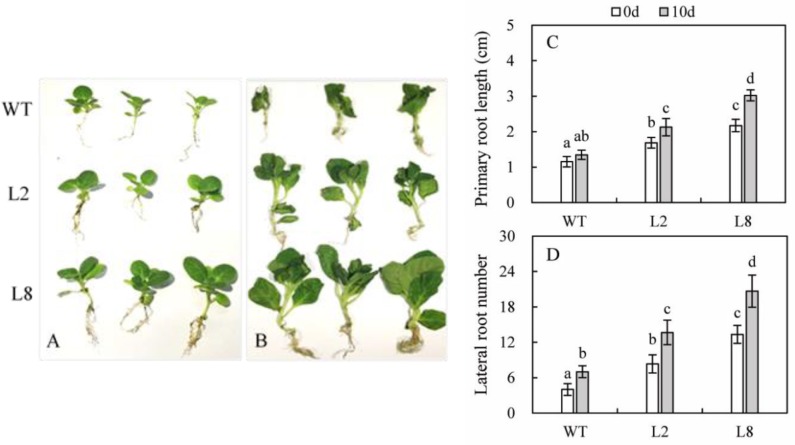
Effect of overexpression of *SoCYP85A1* on root development before and after drought stress. Phenotypes of seedlings at 6-week-old under the normal conditions **(A)** and water loss for 10 d **(B)**. Primary root length **(C)** and lateral root number **(D)** of seedlings at 6-week-old with and without water. Error bars represent SD and values with different letters are significant at *P* < 0.05.

In order to investigate the role of *SoCYP85A1* in the absence of water, seedlings of both WT and the two transgenic lines were subjected to withholding water for 10 days. It could be seen from **Figure 3** that drought caused WT to develop more lateral roots, but length of primary root seemed to be no difference. Besides, leaves of WT curled and wilted seriously, even more of them withered to fall. However, exposed to lack of water, transgenic plants grew better than WT did, whose leaves appeared a little wilting. Furthermore, they developed longer primary root and more lateral roots.

### Overexpression of *SoCYP85A1* Increases *SoCYP85A1* Transcript and CS Accumulation

**FIGURE 4:**
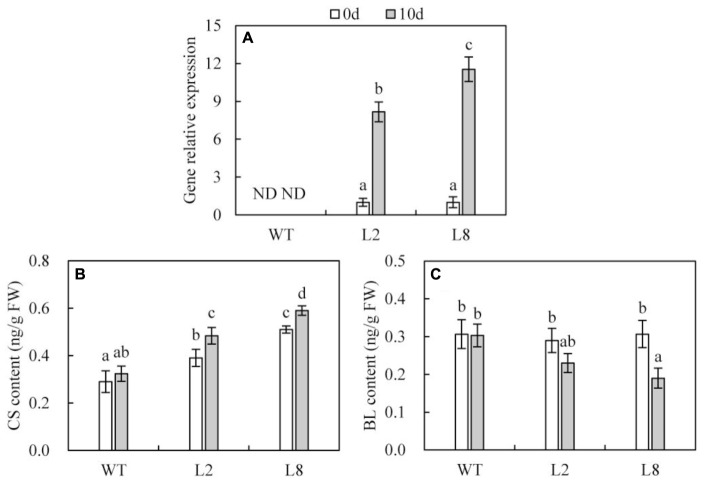
BRs biosynthesis capacity in WT and overexpressing lines (L2 and L8). **(A)** Transcriptional accumulations of the *SoCYP85A1* gene in WT, L2 and L8 before and after 10-day drought stress. The expression level of L2 and L8 at time point 0 was defined as 1, respectively. **(B)** Detection of CS content. **(C)** Detection of BL content. CS, castasterone; BL, brassinolide; FW, fresh weight. The leaves from tobacco plants at 6-week-old stage were sampled for BRs analysis before and after 10-day water loss. Data are the means ± SD of three replicates and values with different letters are significant at *P* < 0.05.

qRT-PCR analysis indicated that the transcription of *SoCYP85A1* was undetectable in WT with or without water. While, exposure to drought stress resulted in the transcriptional expression of *SoCYP85A1* to be enhanced by more than 8 and 11 folds in L2 and L8, respectively (**Figure 4A**).

Quantification of BRs metabolites revealed that overexpression of *SoCYP85A1* induced more CS in transgenic lines than that in WT. Besides, subjected to drought stress, CS in WT just showed a slight increase, while transgenic lines performed a significant rise (**Figure 4B**). In contrast, BL contents had no significant differences between WT and the two transgenic lines before drought treatment. However, drought stress induced BL contents to decrease more notably in transgenic lines than in WT (**Figure 4C**). Additionally, other BRs metabolites including TE (teasterone), TY (typhasterol), 6-deoxoCS (6-deoxo castasterone) and 28-norCS (28-norcastasterone) were all undetectable in both WT and transgenic lines before and after water stress.

### Drought Stress Resulted in Less Accumulation of ABA in Overexpressing *SoCYP85A1* Plants

**FIGURE 5:**
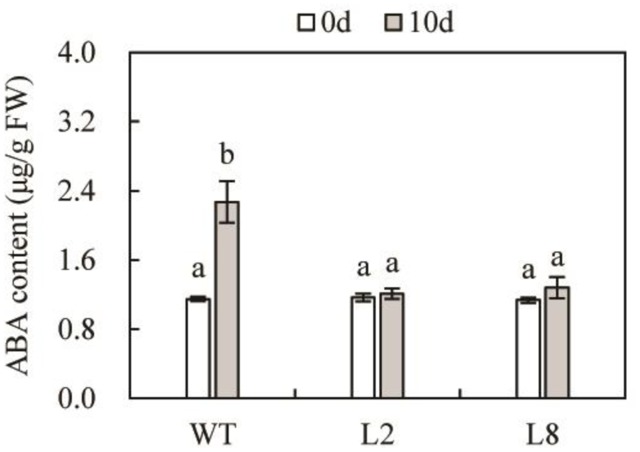
The endogenous contents of ABA in WT and transgenic lines under control and drought treatment. Error bars represent SD and values with different letters are significant at *P* < 0.05.

As is known to all that ABA plays an essential part in plant responses to various abiotic stresses, including drought stress (Lee and Luan, 2012). In order to identify whether transgenic plants enhanced drought tolerance was relevant to ABA accumulation, we determined the concentrations of this compound (**Figure 5**). The data indicated that both WT and the two transgenic lines contained similar level of ABA under the control condition. However, it should be pointed out that drought stress resulted in a significant reduction of ABA content in L2 and L8, compared with WT. ABA accumulation in transgenic lines exhibited a similar level before and after treatment. This result demonstrated that overexpression of *SoCYP85A1* enhanced drought tolerance was not through ABA-dependent pathway.

### Overexpression of *SoCYP85A1* Increases RWC, Proline and Decreases Water Loss Rate, MDA under Drought Treatment

**FIGURE 6:**
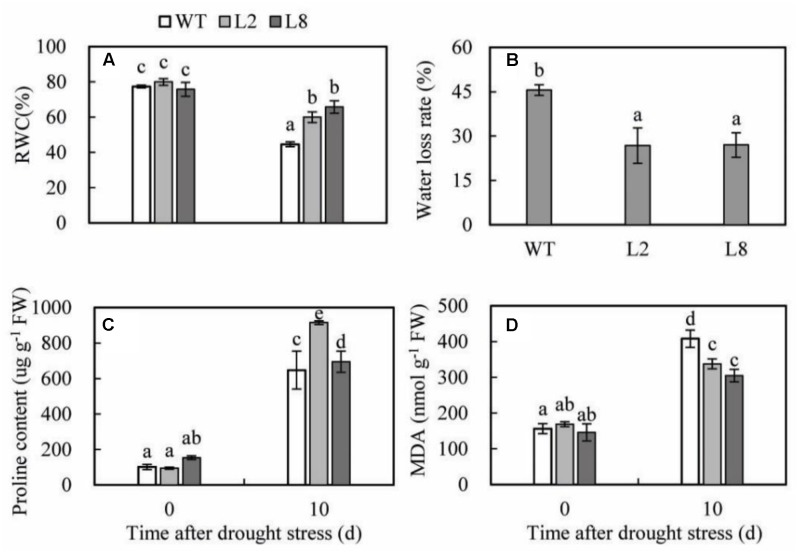
Analysis of the physiological indices in WT and transgenic lines (L2 and L8) under drought stress. Six-week-old seedlings were withheld water for 10 days to measure the value of RWC **(A)**, water loss rate **(B)**, proline content **(C)** and MDA **(D)**. Error bars represent SD and values with different letters are significant at *P* < 0.05.

To investigate the effects of the physiological status caused by overexpression of *SoCYP85A1*, the physiological measurements of RWC, water loss rate, proline, and MDA under drought treatment were conducted. RWC of the two transgenic lines was less reduced than that of WT. Meanwhile, water loss rate presented the opposite profile as RWC. The proline content of the two transgenic lines was higher than that of WT. In addition, MDA was induced lower accumulation in transgenic lines compared with WT under drought stress (**Figure 6**).

### Overexpression of *SoCYP85A1* Increases Antioxidant Enzyme Activities and Decreases the H_2_O_2_ Content under Drought Treatment

**FIGURE 7:**
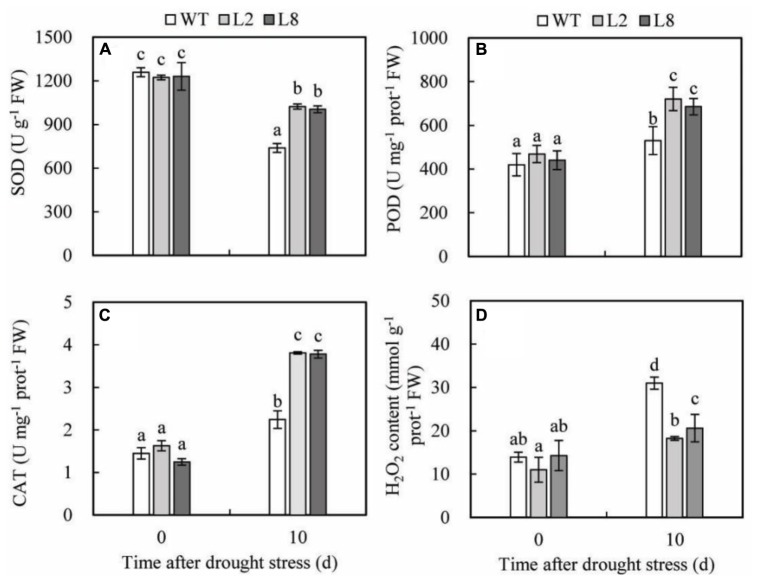
Analysis of three antioxidant enzymes’ activities and H_2_O_2_ accumulation in WT and transgenic lines (L2 and L8) under drought stress. Six-week-old seedlings were withheld water for 10 days to assess SOD **(A)**, POD **(B)**, CAT **(C)**, and H_2_O_2_ **(D)**. Error bars represent SD and values with different letters are significant at *P* < 0.05.

To demonstrate the abilities of scavenging ROS in the transgenic plants, the activities of the three key antioxidant enzymes (CAT, POD, and SOD), which play an important role in scavenging ROS, were detected in WT and transgenic lines before and after treatments as described above. The results presented that before treatments, there were no obvious differences between WT and transgenic plants in activities of CAT, POD, and SOD and the content of H_2_O_2_; however, after treatments, transgenic plants possessed significantly higher activities of CAT than WT did. Drought stress resulted in the increase of POD activity, which showed a remarkable rise in transgenic plants being 1.5 and 1.3 folds of that in WT, respectively. Drought treatment caused a significant decline in SOD activity of WT, which was significantly lower in transgenic plants. SOD activity of L2 and L8 was 1.39 and 1.36 folds of that in WT, respectively (**Figure 7A**). Besides, the H_2_O_2_ accumulation was lower in transgenic plants than in WT (**Figure 7D**), which performed a 41% and 20% increase in WT as compared to L2 and L8, respectively. These results indicated overexpression of *SoCYP85A1* could reduce ROS accumulations by enhancing antioxidant enzyme activities under drought stress.

### *SoCYP85A1* Regulates Expressions of Stress-Relative Genes under Drought Treatment

**FIGURE 8:**
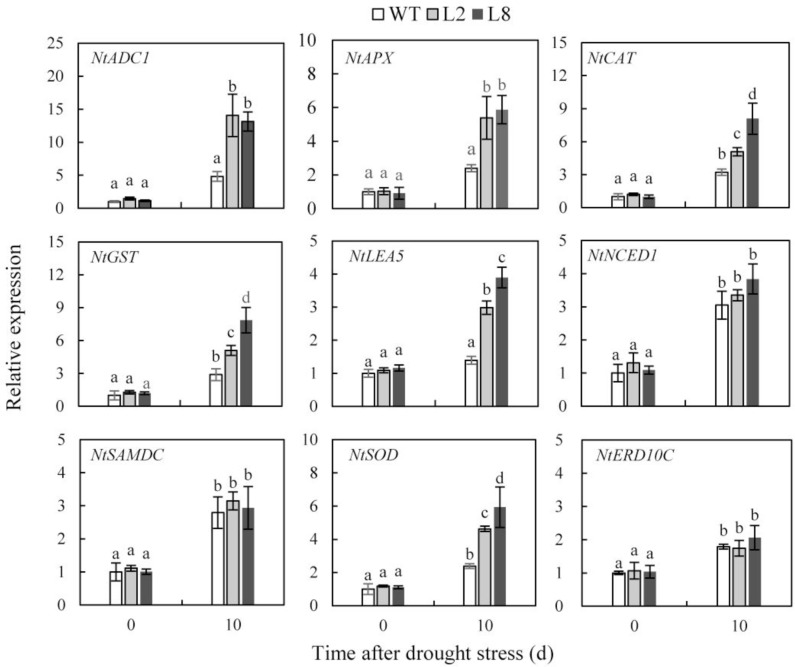
Expression levels of stress-relative genes in WT and transgenic lines (L2 and L8) under drought stress. Six-week-old seedlings were withheld water for 10 days. The tobacco leaves were sampled to extract the total RNA to synthesize cDNA. Expression levels of nine genes were detected. *NtActin* was used as the internal control. Error bars represent SD and values with different letters are significant at *P* < 0.05.

To reveal the molecular mechanism that water loss enhanced drought resistance in transgenic plants, relative quantitative RT-PCR was carried out to investigate expressions of nine genes in both WT and transgenic plants before and after drought stress, which encode key enzymes involved in ABA biosynthesis (*NtNCED1*), polyamine biosynthesis (*NtADC1* and *NtSAMDC*), ROS detoxification (*NtAPX*, *NtCAT*, *NtGST* and *NtSOD*), as well as stress-responsive proteins (*NtERD10C* and *NtLEA5*). Under the condition of withholding water, the transcript levels of *NtAPX*, *NtCAT*, *NtGST*, *NtSOD*, *NtADC1*, and *NtLEA5* were obviously increased in transgenic plants compared with those in WT, whereas the transcript level of *NtNCED1*, *NtSAMDC*, and *NtERD10C* performed a slight rise (**Figure 8**). These results indicated that overexpression of *SoCYP85A1* enhanced drought tolerance by regulating expressions of ROS and stress-responsive related genes.

## Discussion

Most previous researches applied exogenous BRs to analyze, thus the function of the endogenous BRs in the regulation of plant response to drought stress remain unknown and require further analysis (Llanes et al., 2016). Present studies have well documented that CYP85A1 is necessary to catalyze essential reactions for the production of CS, functional studies have been performed for CYP85A1 only in *Arabidopsis*, tomato as well as rice and there are few documentations in spinach. Besides, evidence for the relationships between CYP85A1 proteins and abiotic stresses remains limited. In this work, a gene encoding CYP85A1 from *Spinacia oleracea* was cloned by RACE. A highly conserved Cytochrome P450 domain was observed, indicating that CYP85A1 belongs to the P450 superfamily. Sequence multiple alignment demonstrated that SoCYP85A1 showed high degree of sequence identity with CYP85A1 of other plants retrieved from the database at amino acid level. The phylogenetic tree revealed the relationship between SoCYP85A1 and CYP85A1 from other plants, in which, SoCYP85A1 was closely related to CYP85A1 from *Fagopyrum esculentum* (BAO79854, 81%), *Morus notabilis* (XP_010099314, 80%) and *Arabidopsis thaliana* (NP_851105, 80%). However, current researches about CYP85A1 only limited on biocatalysis or regulations of plant growth and development (Castle et al., 2005; Kim et al., 2005; Pérez-España et al., 2011; Li et al., 2016a), no functional analyses on the relationships between CYP85A1 proteins and abiotic stresses of these three genes have been published to date.

qRT-PCR data showed that *SoCYP85A1* had more abundant transcripts in leaves. However, previous studies indicated that *Arabidopsis CYP85A1* mRNA mainly accumulated in roots (Bancos et al., 2002) or possessed higher transcriptional level in shoots (Shimada et al., 2003). The discrepancy between these results could attribute to the different species and different time of sampling. qRT-PCR analysis revealed that So*CYP85A1* was induced by PEG, NaCl, cold (4°C) and ABA (**Figure 1**), which implicated that *SoCYP85A1* might play important roles in plant abiotic responses. NaCl data showed both, increase and decrease depending on the duration of treatment. We speculate that there might be a feedback mechanism in BRs biosynthesis caused by high salt stress. When the transcription of *SoCYP85A1* increased at the first hour, BRs were induced to produce. However, when BRs accumulated to some extent, the expression of *SoCYP85A1* was suppressed to be lower than that of the control at the third hour. While with the duration of stress, the content of BRs gradually reduced, which relieved the suppression of *SoCYP85A1*. As a result, the transcriptional level of *SoCYP85A1* appeared to increase. This above course did not reciprocate until the transcription of *SoCYP85A1* and the content of BRs reached a dynamic equilibrium. This hypothesis need to be validated on the basis of detecting the content of BRs under the high-salt treatment in our further research.

Compared with the other stresses, drought could cause more abundance of *SoCYP85A1* at the transcriptional level, which prompted us to do further work on exploration of the potential role of this gene for enhancing drought tolerance. Transgenic tobacco were produced via *Agrobacterium* mediated transformation of *SoCYP85A1* under the control of CaMV 35S promoter. The two selected transgenic lines (L2 and L8) exhibited more apparent resistance to drought stress through phenotypic morphology, concomitant with longer primary roots and more lateral roots of transgenic lines than those of WT under drought stress. Apart from drought stress, transgenic seedlings also displayed more tolerance to high-salt tolerance than WT did (data not shown). Our work was consistent with the recent report, in which overexpression of BRs biosynthetic gene *DWF4* increased various stress tolerance (Sahni et al., 2016), implying that BRs biosynthetic genes could enhance plant tolerance to multiple stresses.

Manipulation of BRs biosynthesis has been recognized as a biotechnological target for enhancing crop stress tolerance (Divi and Krishna, 2009). Genetic analysis revealed that loss of CYP85A2 resulted in less BL accumulation and increased drought tolerance in *Arabidopsis* (Northey et al., 2016), which was inconsistent with previous studies that BL increased plant tolerance to drought. These inconsistent results might sound reasonable that any changes in BRs level resulted in differences of plant stress response (Xia et al., 2015). It could be worth noting that due to loss of CYP85A2, BL was sharply decreased, while CS (the precursor of the BL biosynthesis) was accumulated in abundance. The similar result was brought out in our study that transgenic seedlings overexpressing *SoCYP85A1* induced significantly higher CS accumulations performed notably drought tolerance as compared to WT.

There have been increasing evidences that endogenous BRs have been employed to promote plant growth and development, including exogenous application of BRs, inhibitors of BR biosynthesis as well as genetic manipulations of BRs biosynthesis or signaling (Vriet et al., 2012). For instance, overexpression of *CYP85A2* enhanced the growth of *Arabidopsis* with larger rosette leaves and longer petioles compared with WT, because higher level of BL induced by overexpression of CYP85A2 stimulated transgenic plants to hold significant advantages in morphological formation. (Kim et al., 2005). Our result in tobacco were in agreement with this finding. As shown in **Figure 3**, overexpression of *SoCYP85A1* promoted growth and development of seedlings under normal conditions. It should be noticed that exposed to drought stress, this promotion seemed to be notable that transgenic lines possessed larger leaves, longer petioles as well as enhanced root morphology. Therefore, quantification of endogenous BRs was carried out in an effort to examine the influence of *SoCYP85A1* in plant growth and development. The data showed that higher content of CS but not BL was detected in transgenic lines, so we speculated that CS might be a bioactive BRs to trigger the BRs signal transduction pathway to function BRs bioactivities. Besides, the significant increase of CS accumulation in transgenic lines by drought stress indicated that CS might be considered as the drought-stress-induced compound, which was consistent with the finding about Barely BRs mutant (Gruszka et al., 2016). In addition, shoot development was also promoted in transgenic lines, for which the reason might also be that overexpression of *SoCYP85A1* resulted in higher content of CS. CS might be a bioactive BRs to function BRs bioactivities, one of which was stimulation of cell elongation. Therefore, shoot development was enhanced due to the increased content of endogenous CS in transgenic lines.

However, endogenous CS in transgenic *Arabidopsis* was not significantly affected by overexpression of *CYP85A1* (Kim et al., 2005). The reasons for this inconformity might be that there existed different BRs biosynthesis pathways between *Arabidopsis* and tobacco. In the former there are two pathways to biosynthesize CS, part of which is catalyzed to BL. While there is only one pathway to generate CS in the latter, in which trace of CS only turns into 3-epiCS not BL (Suzuki et al., 1995; Fujioka et al., 1997; Hong et al., 2005). Therefore, BL that was detectable in tobacco might be biosynthesized in another pathway since there was a slight change of BL concentrations in WT and *SoCYP85A1* overexpressing seedlings under the normal irrigation. On the other hand, a large accumulation of BL was commonly detected in reproductive organs such as siliques and seeds (Fujioka and Sakurai, 1997), which might indicated that a high level of BL was generated at particular developmental stages. It was well explained that in our result BL concentration was decreased by drought treatment since production of BL was not stimulated at young seedling stage as a result of drought stress. However, exposed to water loss plants generated a high level of CS regulating growth and development and enhancing drought tolerance, suggesting that CS functioned as a BRs bioactivity during seedling growth. Whether CS functions at other periods of tobacco needs to be confirmed. The similar finding had been reported in *Arabidopsis* that CS played an effective role as the bioactive BR that regulated the overall growth and development (Kim et al., 2005).

As is known to all that ABA plays an essential part in plant responses to various abiotic stresses, including drought stress (Lee and Luan, 2012). However, the mechanism that plants coordinate ABA and BRs remains unclear. When grown under optimal watering, both WT and transgenic lines accumulated similar concentrations of ABA. The similar result was also found in tomato that overexpression of *CYP85A1* induced slightly but not significantly higher ABA accumulation over the WT (Li et al., 2016). It was unexpected that drought stress induced a significant increase in the accumulation of ABA in WT but a slight raise in transgenic lines, which exhibited well agreement with the transcriptional level of *NtNECD1* involved in ABA biosynthesis. These results indicated that BRs biosynthesis do not affect the endogenous ABA accumulation under the control condition, while CS functions in negative regulation of ABA under drought stress. It could be an explanation for such regulation that CS and ABA act antagonistically on their target genes in BR signaling pathways particularly when stress responses (Chung et al., 2014).

Then the question was raised what other mechanisms might *CYP85A1* confer the improvement of drought tolerance? To reveal it, we focused our spirit on the physiological differences between WT and transgenic tobacco under drought stress. The results showed that overexpression of *SoCYP85A1* increased RWC and proline, decreased MDA and water loss, which indicated that less oxidative damage occurred in transgenic lines under drought stress. These data were consistent with the recent observation that barely semi-dwarf allelic mutants with an impaired activity of C6-oxidase produced less proline (Janeczko et al., 2016).

H_2_O_2_ level was also detected because it can reflect the degree of damage to plant cell (Miller et al., 2010). Less content of H_2_O_2_ in transgenic lines indicated transgenic lines could scavenge more ROS compared with WT. In order to detoxify ROS induced by drought stress, plants have evolved a complex antioxidant system (Miller et al., 2010), in which, efficient antioxidant enzymes protect plant cell from damages caused by stress through scavenging ROS (Gill and Tuteja, 2010). Among the enzymes, SOD provides the first line of defense against ROS by catalyzing the dismutation of H_2_O_2_, which is then scavenged by the coordinated action of CAT and POD (Blokhina et al., 2003). Our data revealed that activities of SOD, POD, and CAT in transgenic lines were similar as those in WT under normal conditions because ROS remained a low level without stress (Miller et al., 2010). However, after water loss, activities of the three enzymes were significantly higher in transgenic lines than in WT, implying that transgenic lines possessed more efficient detoxifying system when exposed to drought stress. Similar findings have been observed using other plants, such as tobacco, soybean, cucumber, and pistachio (Xu et al., 2011; Radhakrishnan and Lee, 2013; Shu et al., 2013; Kamiab et al., 2014).

To further investigate the mechanism that *SoCYP85A1* enhanced drought tolerance at molecular level, qRT-PCR was carried out to detect transcriptional levels of the four different types of genes before and after drought stress. One type of genes including *NtAPX*, *NtCAT*, and *NtSOD* encodes antioxidant enzymes of POD, CAT and SOD, respectively. The transcriptional amount of the three genes were significantly upregulated in transgenic lines under drought stress. This result was consistent with the data of antioxidant enzyme activities described above, which indicated that the activities of the three enzymes might be regulated by *SoCYP85A1* at transcriptional level. In this case, *SoCYP85A1* might transcriptionally modulate the expression level of the three genes involved in ROS scavenging when it was overexpressed in tobacco.

The next type of genes involved in the polyamines synthesis was *NtADC1* and *NtSAMDC*, which function in adaptive responses to various environmental stresses (Groppa and Benavides, 2008; Jang et al., 2009). Polyamines are thought to play an essential role in scavenging ROS homeostasis (Liu et al., 2015). It was interesting that in our results the transcription of *NtADC1* was induced to be notably enhanced, while *NtSAMDC* remained a similar level after drought stress, suggesting that *NtADC1* played a prominent role in accumulating less ROS in transgenic lines overexpressing *SoCYP85A1*under drought treatment.

Another type of gene was *NtNECD1* that plays an essential role in ABA biosynthesis regulation (Huang et al., 2010). Our result showed that water stress induced expression of *NtNECD1* a notable rise in both WT and transgenic lines. Whereas, it was noticeable that there was not prominent difference in expression level of *NtNECD1* between WT and transgenic lines subjected to water loss, which exhibited an agreement with the quantification of ABA (**Figure 7**), implying that *NtNECD1* was not transcriptionally upregulated by *SoCYP85A1* in tobacco. Our work indicated that overexpression of *SoCYP85A1* enhanced drought tolerance not by ABA-dependent pathway.

The last type of genes containing *NtLEA5* and *NtERD10C* belong to the LEA protein family that protects and stabilizes macromolecules and/or cellular structures during plant stress responses (Xiong and Zhu, 2002; Liu et al., 2013). Our result showed that in the case of water loss, transcriptional level of *NtLEA5* was obviously increased in L2 and L8 compared with that of WT, whereas that of *NtERD10C* changed slightly, which suggested that induction of a higher transcriptional level of *NtLEA5* might be a more effective strategy to resist against drought stress in transgenic lines overexpressing *SoCYP85A1*.

In all, drought treatment led to upregulation of all types of genes in WT and transgenic lines. However, it was worthwhile noticing that mRNA levels of some genes were induced to be higher in transgenic lines than in WT, especially *NtAPX*, *NtCAT*, *NtADC1*, *NtGST* and *NtSOD.* These data demonstrated that the molecular mechanism of enhanced drought tolerance in transgenic lines relied on the inductions of ROS-related genes.

In addition, overexpression of *SoCYP85A1* promoted growth and development of seedlings, especially after drought treatment, transgenic lines possessed larger leaves, longer petioles as well as enhanced root morphology. In this case, it was hard to compare the drought tolerance between WT and transgenic plants with significant different sizes. Therefore, we focused our spirits on physiological indicators. The results indicated that subjected to drought stress transgenic lines possessed higher content of proline, lower accumulation of MDA as well as a more efficient detoxifying system. Furtherly, ABA as another important indicator to measure drought tolerance was quantified in WT and transgenic plants with or without water. It should be kept in mind that ABA content in WT performed a significant increase in the absence of water, while water loss induced a slight rise in ABA accumulation of transgenic lines, suggesting that overexpression of *SoCYP85A1* enhanced drought tolerance was not through ABA-depend pathway. Finally, to investigate the mechanism that *SoCYP85A1* enhanced drought tolerance at molecular level, we carried out qRT-PCR to detect transcriptional levels of stress-responsive genes before and after drought stress. The data showed that expression level of ROS-related genes was notably enhanced in transgenic lines. These above results demonstrated overexpression of *SoCYP85A1* could increase content of proline, reduce accumulation of MDA, establish a more efficient detoxifying system and upregulate transcripts of ROS-related genes to enhance drought tolerance.

## Conclusion

A novel cytochrome p450 gene, *SoCYP85A1*, was upregulated by treatments of PEG, ABA, 4°C and NaCl. Overexpression of *SoCYP85A1* enhanced drought tolerance in transgenic tobacco with increased CS content which promoted root development, accumulated more RWC and proline, less MDA and H_2_O_2_, improved activities of antioxidant enzymes, increased transcriptional expressions of ROS-related and stress-responsive genes under drought stress. Besides, activities of the antioxidant enzymes might be enhanced by *SoCYP85A1* at transcriptional level. In addition, ABA quantification combined with expression level of *NtNECD1* demonstrated that overexpression of *SoCYP85A1* enhanced drought tolerance was not by ABA-dependent pathway. *SoCYP85A1* might involve in plant resistance to drought stress via ABA-independent pathway, which could play the important role in the antioxidant pathway at the transcriptional level. However, the precision mechanisms underlying these results require a further analysis. Therefore, we have already commenced a deeper research, which focuses on transcriptomics and metabolomics studies to explore more target genes regulated by *SoCYP85A1* and more kinds of phytohormone modulated by endogenous BRs in order to establish the relationship among physiological changes, differentially expressed genes and metabolite alterations under drought stress.

## Author Contributions

WS and FD designed and performed part of the experiments; JD, XL, and YF detected BRs, enzyme activities and gene expressions. FD and DL cloned *SoCYP85A1* gene. WS analyzed the data and wrote the manuscript.

## Conflict of Interest Statement

The authors declare that the research was conducted in the absence of any commercial or financial relationships that could be construed as a potential conflict of interest.

## Footnotes

1 www.ncbi.nlm.nih.gov/BLAST/

2 www.expasy.org/protparam/

## Abbreviations

ABA: abscisic acid
BL: brassinolide
BRs: brassinosteroids
CAT: catalase
CS: castasterone
LEA: late embryogenesis abundant
MDA: malondialdehyde
ORF: open reading frame
POD: peroxidase
QRT-PCR: quantitative real-time PCR
ROS: reactive oxygen species
RT-PCR: reverse transcription-PCR
RWC: relative water content
SOD: superoxide dismutase
WT: wild type
