# Supplementary material for: Overexpression of SoCYP85A1, a Spinach Cytochrome p450 Gene in Transgenic Tobacco Enhances Root Development and Drought Stress Tolerance
Source: Front Plant Sci. 2017 Nov 9;8:1909. doi: 10.3389/fpls.2017.01909 (PMC5701648; doi:10.3389/fpls.2017.01909)
Supplement: Supplementary file 1 [file Image_1.PDF]

A

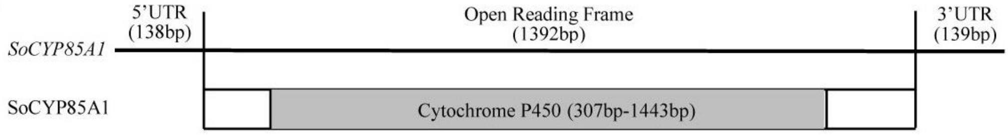
5'UTR (138bp)
Open Reading Frame (1392bp)
3'UTR (139bp)
SoCYP85A1
SoCYP85A1
Cytochrome P450 (307bp-1443bp)

B

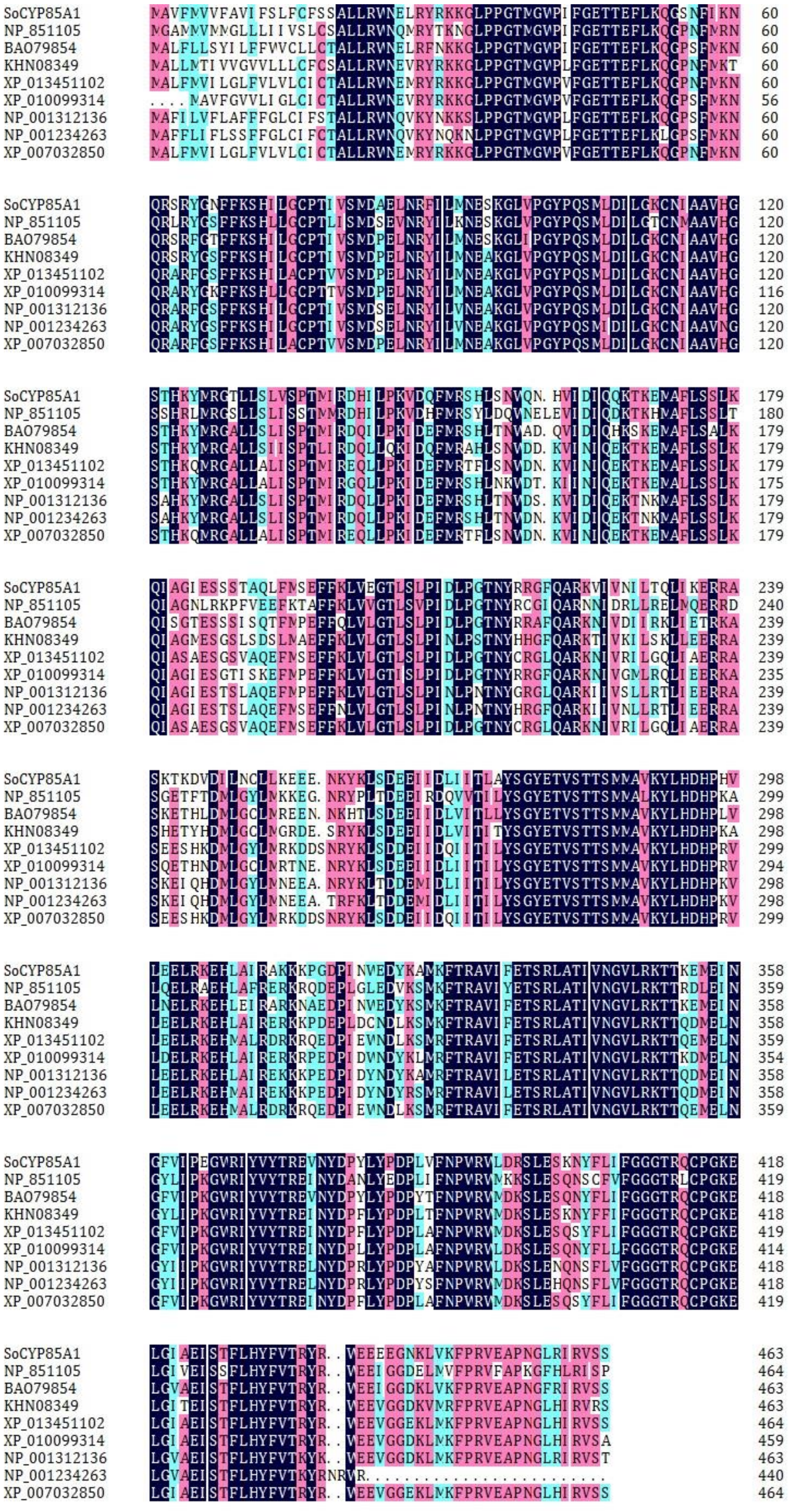
SoCYP85A1
NP_851105
BAO79854
KHN08349
XP_013451102
XP_010099314
NP_001312136
NP_001234263
XP_007032850
463
464
463
463
464
459
463
440
464

C

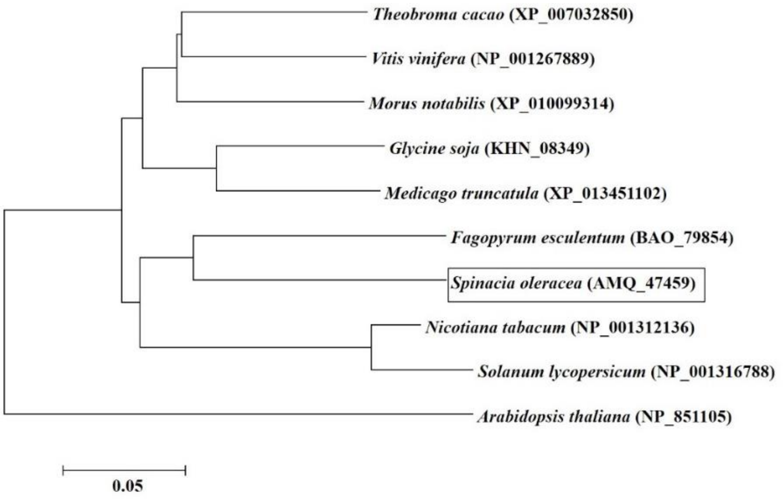

FIGURE 1 | Bioinformatics analysis of the SoCYP85A1 protein. (A) Structure analysis of *SoCYP85A1* containing a conserved domain of cytochrome P450. **(B)** Multiple alignment of SoCYP85A1 with its homologous proteins from other plant species. **(C)** Phylogenetic tree of SoCYP85A1 with its homologous proteins from other plant species.
